# BMP signaling mediates glioma stem cell quiescence and confers treatment resistance in glioblastoma

**DOI:** 10.1038/s41598-019-51270-1

**Published:** 2019-10-10

**Authors:** Rohit Sachdeva, Megan Wu, Kevin Johnson, Hyunsoo Kim, Angela Celebre, Uswa Shahzad, Maya Srikanth Graham, John A. Kessler, Jeffrey H. Chuang, Jason Karamchandani, Markus Bredel, Roel Verhaak, Sunit Das

**Affiliations:** 1 Arthur and Sonia Labatt Brain Tumour Research Centre, Hospital for Sick Kids, Toronto, Ontario Canada; 2 The Jackson Laboratory for Genomic Medicine, Farmington, Connecticut USA; 3 Department of Laboratory Medicine and Pathobiology, University of Toronto, Toronto, Ontario Canada; 4 Institute of Medical Sciences, University of Toronto, Toronto, Ontario Canada; 5 Department of Neurology, Memorial Sloan Kettering, New York City, New York USA; 6 Department of Neurology and Institute for Stem Cell Medicine, Northwestern University, Chicago, Illinois USA; 7 Department of Laboratory Medicine, Montreal Neurological Institute, McGill University, Montreal, Quebec Canada; 8 Department of Radiation Oncology, University of Alabama-Birmingham, Birmingham, Alabama USA; 9 Division of Neurosurgery, Li Ka Shing Knowledge Institute, St. Michael’s Hospital, University of Toronto, Toronto, Ontario Canada

**Keywords:** CNS cancer, Cancer stem cells

## Abstract

Despite advances in therapy, glioblastoma remains an incurable disease with a dismal prognosis. Recent studies have implicated cancer stem cells within glioblastoma (glioma stem cells, GSCs) as mediators of therapeutic resistance and tumor progression. In this study, we investigated the role of the transforming growth factor-β (TGF-β) superfamily, which has been found to play an integral role in the maintenance of stem cell homeostasis within multiple stem cell systems, as a mediator of stem-like cells in glioblastoma. We find that BMP and TGF-β signaling define divergent molecular and functional identities in glioblastoma, and mark relatively quiescent and proliferative GSCs, respectively. Treatment of GSCs with BMP inhibits cell proliferation, but does not abrogate their stem-ness, as measured by self-renewal and tumorigencity. Further, BMP pathway activation confers relative resistance to radiation and temozolomide chemotherapy. Our findings define a quiescent cancer stem cell population in glioblastoma that may be a cellular reservoir for tumor recurrence following cytotoxic therapy.

## Introduction

Glioblastoma is the most prevalent and aggressive malignant brain tumour in adults, with a median survival following multi-modality therapy of 14.6 months^1^. Recent studies have implicated cancer stem cells within glioblastoma (glioma stem-like cells, GSCs) as mediators of tumor growth, therapeutic resistance and tumor progression^2–5^. GSCs also retain the genetic features of parental tumors, suggesting they are a faithful model system for human glioblastoma^6,7^.

Examination of other physiologic and cancer stem cell systems has shown that these cells are phenotypically dynamic, and that the functional plasticity of these cells is modulated by both cell-intrinsic and cell-extrinsic factors, including signaling within the stem cell microenvironment^8,9^. For example, neural stem cells (NSCs) within the subventricular zone and hair follicle stem cells (HFSCs) within the hair follicle bulge have been shown to transition between quiescent and activated states^10,11^. Further, transcriptional heterogeneity has been proposed as a mechanism to balance self-renewal and differentiation in NSCs, HFSCs, and hematopoietic stem cells^12–18^. The epigenetic processes underlying cell fate specification in GSCs are less well understood.

One primary agent of cell fate specification in multiple developing and adult stem cell systems is the family of inhibitor of DNA-binding genes, ID1-ID4. During development, the ID genes are actively expressed in stem and progenitor cells to support proliferation and inhibit differentiation, whereas the ID genes are repressed upon lineage commitment and differentiation^19,20^. In addition to its role in hair follicle stem cell physiology, ID1 has been postulated to mark the bona fide adult neural stem cell^21,22^. The ID family member ID1 has also been found to be dysregulated in cancer and to direct multiple hallmarks of cancer, such as cell growth and survival, invasion and migration, and angiogenesis^23–26^. In glioblastoma, ID1 knockdown in a mouse xenograft model impairs cell invasion and thereby results in increased overall survival^27,28^. In colon cancer, knockdown of ID1 and ID3 have been shown to impair self-renewal of colon cancer tumor initiating cells, reduce tumor growth and enhance sensitivity to chemotherapy^26^. Interestingly, ID1 ablation in a mouse transgenic glioblastoma model failed to render these cells non-tumorigenic; in fact, ID1 loss appeared to enhance tumor growth and shorten latency to end point^29^.

There is growing evidence to suggest that the superfamily of transforming growth factor-βs (TGF-βs) play an integral role in the maintenance of stem cell homeostasis through their effects on ID1 expression within multiple stem cell systems^30^. In the hair follicle stem cell niche, dampening of a repressive bone morphogenetic protein (BMP) signal by TGF-β2 is necessary for hair follicle stem cells to transition from the telogen (quiescent) to anagen (activated) state^31^, a process that requires the suppression of BMP-mediated expression of ID1^32^. Similarly, ID1 expression restrains proliferation and fate commitment in hematopoietic stem cells (HSCs), and is required for HSC repopulation and maintenance^33,34^.

We postulated that BMP and TGF-β signaling could have an analogous role for human GSCs through their effect on ID1. Here, we report that BMP and TGF-β signaling define divergent molecular and functional identities in glioblastoma, and mark relatively quiescent and proliferative GSCs, respectively. Treatment of GSCs with BMP inhibits cell proliferation, but does not abrogate their stem-ness, as measured by self-renewal and tumorigencity. Further, BMP pathway activation confers relative resistance to radiation and temozolomide chemotherapy. The development of temozolomide resistance results in selection of cells with prolonged cell-cycle time and latency of tumor formation. Finally, we find that p21 mediates the effect of BMP signaling on glioma cell proliferation and chemotherapeutic resistance. Our findings define a quiescent cancer stem cell population in glioblastoma that may be a potentially targetable cellular reservoir for tumor recurrence following cytotoxic therapy.

## Results

### BMP and TGF-β signaling define divergent molecular identities in glioblastoma

**Figure 1:**
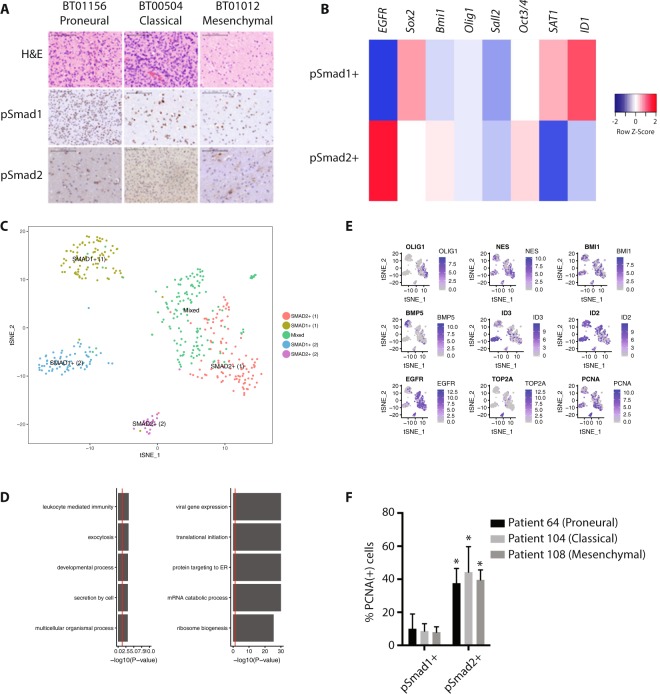
BMP and TGF-β signaling define divergent molecular identities in glioblastoma. (**A**) H&E and IHC for pSmad1 (BMP) and pSmad2 (TGF-β) in human glioblastoma surgical specimens (n = 9). (**B**) Single-cell analysis of pSmad1- and pSmad2-positive cells isolated by laser capture microscopy. (**C**) Unsupervised clustering of single-cell glioblastoma RNA-seq data using the most variable genes. Cell identity was determined using cell markers for pSmad1 and pSmad2-positive glioma cells. (**D**) Log-normalized gene expression for selected genes is shown for the SMAD1(+) and SMAD2(+) glioma cell populations defined in 1 C. (**E**) Gene set enrichment analysis for genes upregulated in SMAD1(+) (left) and SMAD2(+) (right) glioma cell populations identified by analysis of single-cell glioblastoma RNA-seq data. (**F**) Quantification of PCNA-expression in pSmad1- and pSmad2-positive cells in human glioblastoma surgical specimens (n = 4, 15 HPF/specimen; *p < 0.05).

To determine if the BMP and TGF-β pathways are activated in glioblastoma cells in the tumor microenvironment, we performed immunohistochemistry on human glioblastoma specimens (n = 9) for the activated nuclear R-SMADs, pSmad1 (BMP) and pSmad2 (TGF-β). We found evidence of nuclear pSmad1 and pSmad2 staining in all molecular subtypes that we examined (Fig. 1A). To confirm that BMP and TGF-β activation was present in glioma cells rather than stromal non-neoplastic cells^35^, we repeated these studies in a glioblastoma possessing an R132H IDH1 mutation, in which glioma cells can be differentiated from non-glioma cells by the presence of the IDH1 R132H mutation. Both pSmad1-positive and pSmad2-positive cell populations were IDH1-mutant, confirming their identity as glioma cells (Supplementary Fig. 1A,B).

As previous reports have shown that BMP signaling directs astroglial differentiation in GSCs^36–38^, we expected that pSmad2-positive glioma cells would be enriched in stem cell markers, such as Sox2 and nestin, compared to pSmad1-positive glioma cells. Surprisingly, single-cell qRT-PCR analysis of pSmad1- and pSmad2-positive cells isolated by laser capture microscopy showed that both populations of cells expressed markers of stem-ness, such as OLIG1, SALL2, OCT3/4, and SOX2, compared to normal brain. pSmad1-positive cells expressed high levels of SAT1 and ID1, while pSmad2-positive cells expressed high levels of EGFR (Fig. 1B).

We then sought to determine the phenotypic identity of pSmad1-positive and pSmad2-positive glioma cells using publicly available single-cell glioblastoma RNA-seq (scRNAseq) data^39,40^. First, we performed unsupervised clustering of the scRNAseq dataset (n = 430 single cells, 5 patients) to define single-cell clusters. We then ascribed cell identity to these clusters using the marker gene information identified by our single-cell qRT-PCR analysis described above. This analysis identified two SMAD1(+) and two SMAD2(+) cell populations, as well as one “mixed” cell population (Fig. 1C). Overall, we observed 956 genes differentially expressed between the SMAD1(+) and SMAD2(+) glioma cell populations (Bonferroni-adjusted *P* < 0.05); 671 genes were upregulated in SMAD2+ cells, while 285 were upregulated in SMAD1(+) cells. Gene set enrichment analysis identified gene pathways involved in cell replication and metabolism as active in SMAD2(+) glioma cells, while SMAD1(+) glioma cells showed upregulation of gene associated with cellular immune response, developmental processes, and exocytosis, suggestive of a drug-resistant population (Fig. 1D). SMAD2+ glioma cells were highly enriched for the DNA polymerase factor, PCNA, consistent with the conclusion that this cell population housed the majority of dividing cells (Fig. 1E).

To interrogate the conclusion that SMAD2(+) identified the proliferation fraction of glioblastoma cells, we performed double-label immunohistochemistry for pSmad1 or pSmad2 and PCNA in human glioblastoma surgical specimens (n = 4, 15 HPF/specimen). Consistent with our scRNAseq analysis, we found that only 10.1 ± 8.9% of pSmad1-positive cells were also PCNA-positive, compared to 37.6 ± 8.9% of pSmad2-positive cells, which also labeled for PCNA (Fig. 1F; p < 0.05).

### Glioma stem cell phenotype is modulated by treatment with BMP4 or TGF-β1

**Figure 2:**
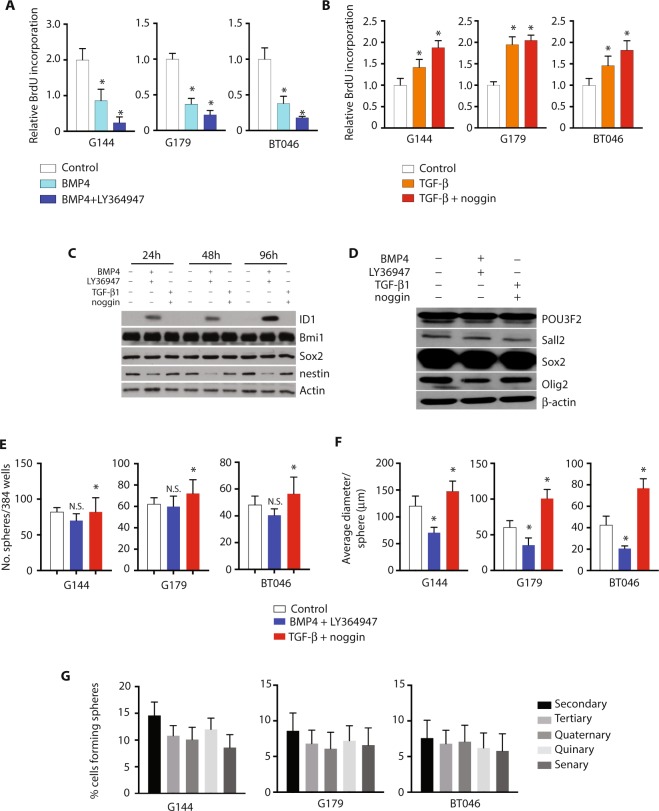
Glioma stem cell phenotype is modulated by treatment with BMP4 or TGF-β1. (**A**) Relative BrdU incorporation in G144, G179, and BT046 cells, treated with BMP4 or BMP and LY364947, compared to control (n = 3, *p < 0.01). (**B**) Relative BrdU incorporation in G144, G179, and BT046 cells, treated with TGF-β1 or TGF-β1 and noggin, compared to control (n = 3, *p < 0.05). (**C**) Western blot analysis for the stem cell markers ID1, Bmi1, Sox2, and nestin, following treatment of BT046 with BMP4, LY364947, TGF-β1, or noggin. (**D**) Western blot analysis for POU3F2, Sall2, Sox2, and Olig2, in BT046 cells following treatment with BMP4, LY364947, TGF-β1, or noggin. (**E**) Sphere formation by BT046 following treatment with BMP4 and LY364947 or TGF-β1 and noggin, compared to control (n = 3, *p < 0.05). (**F**) Average diameter of spheres formed by BT046 following treatment with BMP4 and LY364947 or TGF-β1 and noggin, compared to control (n = 3, *p < 0.01). (**G**) Serial passaging of G144, G179, and BT046 cells treated with BMP4 (n = 3, N.S.).

To determine if differences in cytokine signaling could in themselves account for the phenotypic divergence seen *in situ*, we examined the effect of TGF-β and BMP signaling on cell phenotype in three GSC lines *in vitro*. Treatment of GSCs with recombinant BMP4 or TGF-β1 resulted in a highly stereotypical and reproducible expression profile (Supplementary Fig. 2). BMP4-treated GSCs showed a greater than 2-fold decrease in cell proliferation (Fig. 2A; n = 3, p < 0.01), an effect that was significantly amplified by concomitant exposure to the TGF-β inhibitor, LY364947. Treatment with LY36494 alone had no significant effect on cell proliferation. Conversely, exposure of GSCs to TGF-β1 resulted in an increase in cell proliferation, with minimal amplification following concomitant exposure to the BMP antagonist, noggin (Fig. 2B; n = 3, p < 0.05). Our findings suggest that GSCs in proliferative culture secrete TGF-β. Indeed, analysis of the culture media using ELISA showed high levels of TGF-β1 and TGF-β2, but not BMP1, BMP2, BMP4, or BMP7, under baseline culture conditions (Supplementary Fig. 3A–F).

Consistent with our *in situ* findings, neither BMP4 nor TGF-β1 significantly affected expression of the neural stem cell markers, Sox2 and Bmi1, in GSCs (Fig. 2C). Of note, we found that ID1 expression in GSCs was induced by treatment with BMP4 rather than TGF-β, in contrast to indirect evidence from others that ID1 is a downstream effector of TGF-β1 in glioblastoma^41^. We found no difference in expression of the core GSC transcription factors Oct3/4, Sox2, Sall2, and Olig1, between GSCs treated with BMP4 or TGF-β1 (Fig. 2D). Further, BMP- and TGF-β1-treated GSCs could not be discriminated on the basis of expression of the Bernstein GSC panel of nineteen tumor-propagating cell (TPC)-specific transcription factors (Supplementary Fig. 4A)^39,40^. Consistently, TPCs share a BMP- and TGF-β-responsive target gene expression profile that is distinct from that of differentiated glioma cells (DGCs; Supplementary Fig. 4B). These findings suggested to us that BMP and TGF-β1 both modulate but do not abolish the GSC phenotype, and might instead control the transition of GSCs from a quiescent to a proliferative state.

### BMP4 inhibits but does not abrogate GSC self- renewal and tumorigenicity

To test the hypothesis that BMP modulates but does not abolish the GSC phenotype, we examined the effect of BMP or TGF-β exposure on GSC self-renewal using the neurosphere assay system. Exposure of GSCs to TGF-β1 resulted in increased sphere formation by GSCs grown at clonal density (Fig. 2E; n = 3, p < 0.05), consistent with a positive effect on self-renewal, and also resulted in an increase in average sphere diameter, consistent with an increase in cell proliferation (Fig. 2F; n = 3, p < 0.01). BMP4 exposure diminished but did not abrogate sphere formation (Fig. 2E, n = 3, N.S.), and resulted in attenuation in gliomasphere size (Fig. 2F; n = 3, p < 0.05. Further, BMP4-treated GSCs continued to form spheres with serial passaging (Fig. 2G; n = 3, N.S.), indicating that these cells remained capable of self-renewal.

**Figure 3:**
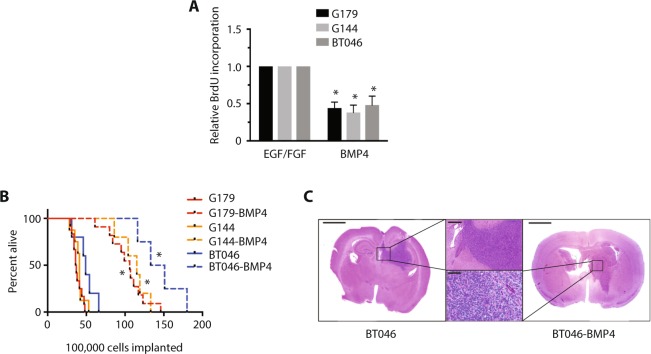
BMP-treated GSCs are tumorigenic with prolonged latency. (**A**) Relative BrdU incorporation in G179, G144, and BT046 cells, cultured in GSC media (EGF/FGF) or GSC media with BMP4 (n = 3, *p < 0.01). (**B**) Kaplan-Meier curve for survival following intracranial transplantation of NSG mice with G179, G144, or BT046 GSCs, with or without BMP4 pre-treatment (n = 5 per group; *p < 0.01). (**C**) Representative H&E staining of NSG mice at endpoint following transplantation with BT046 (left, upper panel) and BT046-BMP (right, lower panel). Scale bar represents 2000 μm (whole mount) or 500 μm (inlay).

We then sought to determine if BMP exposure affected GSC tumourigenic potential following orthotopic transplantation. GSCs were cultured in normal media supplemented with EGF and FGF (BT062508, BT051010, BT030909) or in media without factors and with BMP4 (BT062508-BMP4, BT051010-BMP4, BT030909-BMP4) for five days. As would be predicted by our prior studies in other GSC lines, BMP4-treated cells showed a significant decrease in proliferation, as determined by BrdU incorporation (Fig. 3A; n = 3, p < 0.01), and increased ID1 expression, with no significant change in Sox2 or nestin expression. Control and BMP4-treated GSCs were then dissociated and transplanted into the right frontal striatum of immunocompromised (NOD *scid*) mice (n = 5/group). As in mice transplanted with control GSCs, mice transplanted with BMP4-treated GSCs developed high-grade gliomas, though with prolonged latencies to tumour formation, compared to control cells (Fig. 3B; p < 0.01). Tumors formed by GSCs with or without BMP4 pre-treatment were histologically indistinguishable on analysis by a neuropathologist (J.K., Fig. 3C). These findings are consistent with a previous report using a transgenic mouse model that ablation of ID1 does not result in loss of tumorigenicity, but actually leads to shortened tumor latency following transplantation^29^.

### ID1 identifies a long-term label retaining cell in glioblastoma

Our *in vitro* findings that BMP signaling induces quiescence in GSCs are in conflict with previous reports that BMP directs GSCs toward a terminally differentiated astroglial cell fate^36^. To distinguish between these two opposing hypotheses, we performed two long-term label retaining cell (LRC) assay studies in a glioblastoma xenograft model^42^. The LRC assay exploits the dynamics of integration and retention of a tagged synthetic nucleoside into the DNA through the cell cycle. The tag will only be found in cells that have undergone DNA replication during a period overlapping that of delivery of the synthetic nucleoside. Further, the signal will diminish as that cell undergoes further cell divisions. For our experiments, we employed the nucleoside analog 5-ethynyl-2′-deoxyuridine, marked with a fluorescent tag (EdU-FITC; Invitrogen).

**Figure 4:**
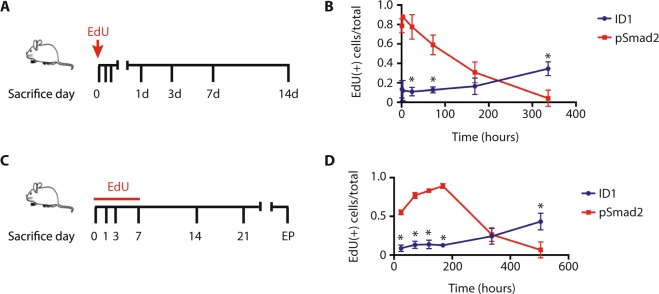
ID1 identifies a long-term label retaining cell in glioblastoma. (**A**) Schematic of chase following a single intraperitoneal injection of EdU in NSG mice harboring BT046 intracranial xenograft tumors (n = 3/time point). (**B**) Quantification of relative frequency of EdU positivity in ID1- and pSmad2-positive cells (*p < 0.001). (**C**) Schematic of chase following a 7-day continuous load with EdU in NSG mice harboring BT046 intracranial xenograft tumors. (**D**) Quantification of relative frequency of EdU positivity in ID1- and pSmad2-positive cells (*p < 0.001).

We first sought to determine if BMP-activated glioma cells differ in their likelihood to enter the cell cycle compared with the TGF-β-activated glioma cells that make up the most of the tumor bulk. To do so, mice harboring a mature glioblastoma xenograft were administered a single dose of EdU through intraperitoneal injection and then sacrificed at successive time points thereafter (Fig. 4A; n = 3/time point). In this paradigm, EdU labeling will be limited to cells entering or within the cell cycle at the time of its administration. The label will rapidly decay in cells that go on to re-enter the cell cycle. ID1 was used as a proxy for BMP activation, while we used phosphorylated Smad2 as a marker of TGF-β activation. At 1 hour following EdU administration, 13.4 ± 5.3% of ID1-positive cells were also EdU positive (Fig. 3B), compared to 78.8 ± 21.4% of pSmad2-positive cells (Fig. 4B; p < 0.001). By 14 days, 34.6 ± 3.7% of ID1-positive cells were EdU-positive, compared to 4.1 ± 0.5% of pSmad2-positive cells (p < 0.001). Time-point analysis showed rapid decay of EdU signal from pSmad2-positive cells, with no significant loss of signal from ID1-positive cells, consistent with a divergence in cell cycle entry between BMP-activated and TGF-β-activated glioma cells.

We then sought to determine if BMP-activated glioma cells are LRCs. To do so, we employed a paradigm in which mice were administered EdU for 7 days, which should result in labeling of most if not all mitotically active cells within the tumour, followed by a long-term chase (Fig. 4C; n = 3/time point). Mice were sacrificed and brain tissues collected on days 7, 14, and 21, following completion of EdU administration, or at endpoint. The frequency of Edu/ID1 and Edu/pSmad2 double-positive cells was quantified from primary tumor sections throughout the labeling and chase. At the initial time-point, we found that 8.8 ± 4.1% of ID1-positive cells were EdU-positive, compared to 55.0 ± 5.5% of pSmad2-positive cells (Fig. 4D; p < 0.001)). By 21 days, 43.3 ± 10.8% of ID1-positive cells were EdU-positive, compared to 6.7 ± 0.4% of pSmad2-positive cells (p < 0.001). Time-point analysis demonstrated that EdU signal steadily reduced in the pSmad2-positive population. Surprisingly, the percentage of ID1-positive cells that were also EdU-positive increased during the chase, suggesting that some of the cells in this population may have transitioned to no longer express ID1. Taken together, these studies demonstrate that BMP pathway activation demarcates a slow cycling, long-term label retaining cell population in glioblastoma.

### pSmad1 identifies a pool of treatment-resistant glioma cells

**Figure 5:**
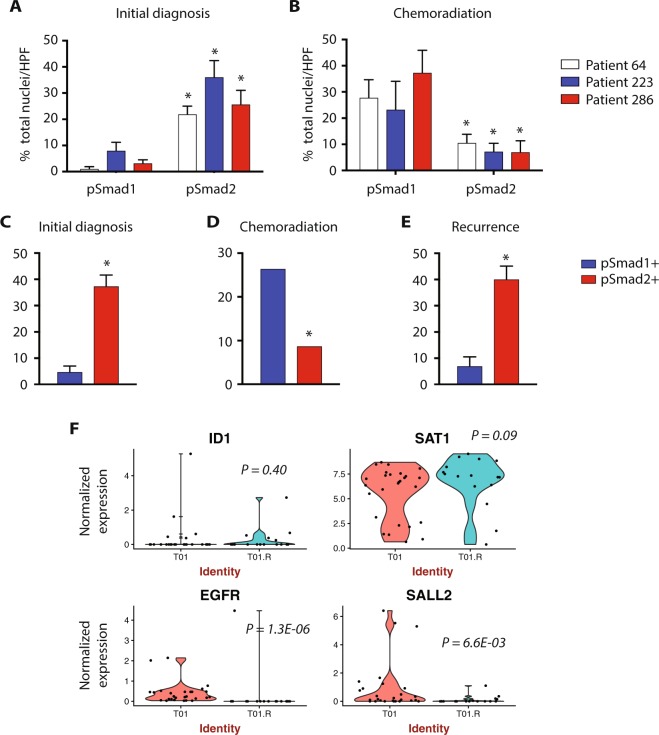
pSmad1 identifies a pool of treatment-resistant glioma cells. (**A**) Quantification of pSmad1- and pSmad2-immunopositivity in glioblastoma specimens harvested from patient 64, patient 223, and patient 286, at initial diagnosis (*p < 0.01). (**B**) Quantification of pSmad1- and pSmad2-immunopositivity in glioblastoma specimens harvested from patient 64, patient 223, and patient 286, during chemoradiation with temozolomide (*p < 0.01). (**C**–**E**) Quantification of pSmad1- and pSmad2-immunopositivity in glioblastoma specimens harvested from a single patient at initial diagnosis, during chemoradiation with temozolomide, and at frank recurrence (*p < 0.01). (**F**) Differential expression analysis (Wilcoxon-rank sum test) of single cell glioma RNA-seq data from a single patient at initial diagnosis (T01) and tumor recurrence (T01.R) (n = 43 single cells, 27 cells at initial diagnosis and 16 cells at recurrence).

LTR cells are thought to constitute a treatment-resistant cell population in multiple hematopoietic and solid cancer subtypes, including acute lymphoblastic leukemia^43^, pancreatic cancer^44^, colorectal cancer^45^, and breast cancer^46^. To determine if BMP-activated (pSmad1+) glioma cells could constitute a treatment-resistant cell population in glioblastoma, we performed IHC from paired human glioblastoma specimens harvested at initial diagnosis and immediately following radiation and temozolomide chemotherapy. As clinical practice does not conventionally allow for harvest immediately following completion of adjuvant therapy, our analysis was limited to three patients in whom tissue harvest at this point was driven by concern of early relapse. At both time points, we analyzed regions corresponding to sites of Gadolinium contrast enhancement on magnetic resonance imaging (Supplementary Fig. 5A–C). At initial diagnosis, pSmad2+ cells constituted the majority of identified tumor cells, while pSmad1+ cells constituted a small minority, in all three patients (Fig. 5A; p < 0.01). Conversely, analysis of biopsy specimens harvested at the immediate conclusion of radiation and temozolomide chemotherapy showed a significant increase in the pSmad1+ cell population, as well as a significant decrease in the pSmad2+ cell population (Fig. 5B; p < 0.01).

We were then able to identify one patient in whom paired tumor samples were available from the time of initial diagnosis, immediately following completion of adjuvant therapy, and at frank radiographic recurrence (Supplementary Fig. 6). In this single patient, analysis at initial diagnosis showed that the majority of glioma cells were marked by pSmad2, while only a minority of cells were found to be pSmad1-positive (Fig. 5C; p < 0.01). As found in our first cohort, analysis of analysis of biopsy specimens harvested at the immediate conclusion of radiation and temozolomide chemotherapy showed a significant increase in the pSmad1+ cell population, as well as a significant decrease in the pSmad2+ cell population (Fig. 5D; p < 0.01). Interestingly, analysis of tumor tissue harvested at the time of frank recurrence once again showed an abundance of pSmad2+ cells (Fig. 5E; p < 0.01).

Finally, we analyzed publicly available scRNA data from a primary glioblastoma and matched recurrence (n = 43 single cells, 27 cells at initial diagnosis and 16 cells at recurrence)^47^. While the sample contained too few cells to allow for meaningful unsupervised clustering, differential expression of analysis of cells at recurrence revealed a trend toward enrichment of the SMAD1(+) glioma cells markers, ID1 and SAT1, and a statistically significant loss of the SMAD2(+) glioma cell markers, EGFR and SALL2 (Fig. 5F). These data suggest that pSmad1+ cells are enriched by radiation and temozolomide chemotherapy, and could serve as a reservoir for tumor recurrence.

### Activation of the BMP signaling pathway protects glioma cells from cytotoxic injury

**Figure 6:**
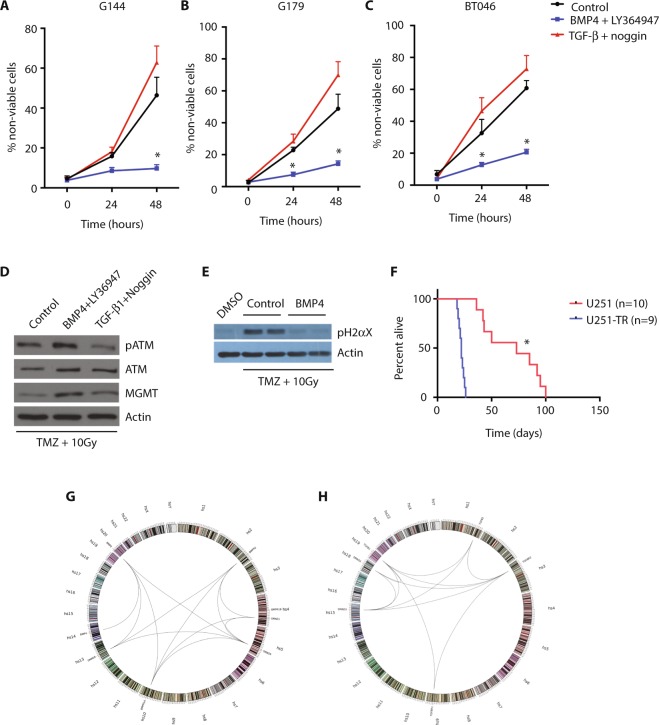
Activation of the BMP signaling pathway protects glioma cells from cytotoxic injury. (**A**) Cell viability following treatment of G144 cells with temozolomide and radiation, with BMP4 and LY364947 or TGF-β1 and noggin, compared to control (n = 3, *p < 0.05). (**B**) Cell viability following treatment of G179 cells with temozolomide and radiation, with BMP4 and LY364947 or TGF-β1 and noggin, compared to control (n = 3, *p < 0.05). (**C**) Cell viability following treatment of BT046 cells with temozolomide and radiation, with BMP4 and LY364947 or TGF-β1 and noggin, compared to control (n = 3, *p < 0.05). (**D**) Western blot analysis for phosphorylated ATM (pATM), ATM, and MGMT, in BT046 cells treated with temozolomide and radiation, with BMP4 and LY364947 or TGF-β1 and noggin, compared to control. (**E**) Western blot analysis for phosphorylated H2αX in BT046 cells treated with temozolomide and radiation, with BMP4 and LY364947 or TGF-β1 and noggin, compared to control. (**F**) Kaplan-Meier curve for survival in NSG mice following intracranial transplantation with U251 or U251-TR cells (*p < 0.01). (**G**) Circos plot of expression data generated by microarray analysis of U251 cells. (**H**) Circos plot of expression data generated by microarray analysis of U251-TR cells.

To examine if BMP activation could account for treatment resistance in glioblastoma cells, we performed *in vitro* survival studies in three GSC lines (G144, G179, BT046) exposed to a five-day course of temozolomide (25 μM) and concomitant radiation (10 Gy/5 sessions) following treatment with BMP or TGF-b pathway agonists. As expected, treatment of GSCs with temozolomide and radiation resulted in substantial cell death. The cytotoxic effect of temozolomide and radiation was significantly attenuated by pre-treatment of GSCs with BMP4, while pre-treatment with TGF-β significantly increased the amount of cell death that occurred with cytotoxic therapy (Fig. 6A–C; n = 3, p < 0.05). Treatment of GSCs with BMP4 resulted in increased expression of the DNA repair protein, O^6^-methylguanine DNA methyltransferase (MGMT), as well as increased phospho-activation of the DNA repair modulator, ATM serine/threonine kinase (ATM) (Fig. 6D). Finally, treatment of GSCs with BMP4 resulted in decreased expression of the DNA damage mark, gamma-H2AX phosphorylation (pH2AX), in GSCs following exposure to temozolomide and radiation (Fig. 6E).

Disease relapse in acute myeloid leukemia after conventional chemotherapy is thought to be caused by quiescent leukemic stem cells, which are able to survive cytotoxic therapy and drive disease recurrence^48^. We hypothesized that treatment resistance in glioblastoma could also be mediated by relative cell quiescence. To test this hypothesis, we developed temozolomide-resistant cell lines using U251 and ID1^−/−^.U251.1/2 cells. The parental cells were cultured with a biweekly increasing dose of temozolomide, starting at 10 µM to a maximum dose of 200 µM. A selected population of U251 cells survived this treatment (U251 temozolomide-resistant cell line; U251.TR) and were definably temozolomide resistant, as determined by IC50 and TMZ sensitivity assay for viability following treatment (Supplementary Fig. 7A,B). Compared to parental U251 cells, U251.TR cells had no significant change in proliferation rate *in vitro* (Supplementary Fig. 7C). However, while U251.TR tumors were histologically indistinguishable from tumors formed by their parental counterparts (Supplementary Fig. 7D,E), they had an increased latency of tumor formation following intracranial implantation in immunocompromised mice (Fig. 6F; p < 0.01). Further, expression analysis showed that U251.TR cells exhibit a BMP activation signature (Fig. 6G), in contrast to parental U251 cells, which exhibit a TGF-β activation signature (Fig. 6H).

### p21 mediates the effects of BMP4 as an anti-proliferative and cytoprotective signal in glioblastoma

**Figure 7:**
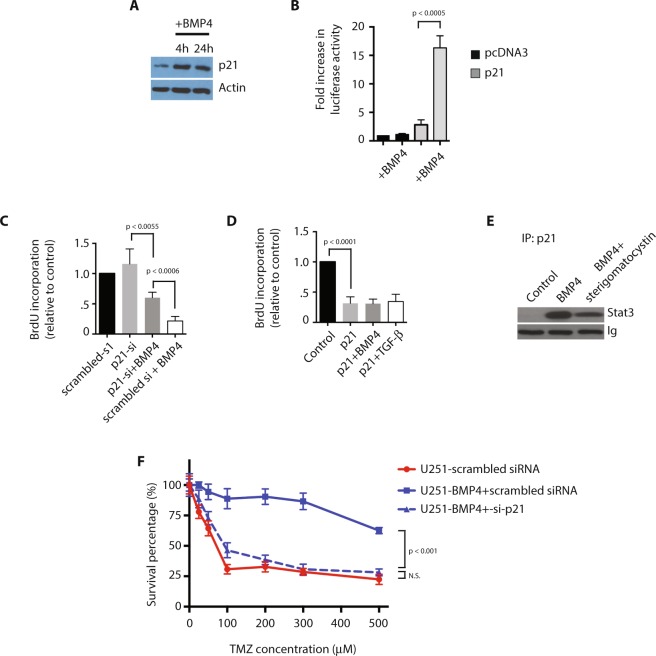
p21 mediates the effects of BMP4 as an anti-proliferative and cytoprotective signal in glioblastoma. All experiments were performed using the U251 glioblastoma cell line. (**A**) Western blot analysis for p21 following treatment with BMP4. (**B**) Luciferase assay using a p21 promoter reporter in U251 cells with and without treatment with BMP4 (n = 3). (**C**) Cell proliferation as measured by BrdU incorporation in U251 cells treated with siRNA against p21 with or without BMP4 (n = 3). (**D**) Cell proliferation as measured by BrdU incorporation in U251 cells transduced with a p21 overexpression vector and treated with BMP4 or TGF-β1 (n = 3). (**E**) Western blot analysis for Stat3 following immunoprecipitation for p21 in U251 cells following treatment with BMP4 with or without sterigomatocystin. (**F**) IC50 curves for temozolomide in U251 cells treated with BMP and scrambled siRNA or BMP and p21 siRNA, compared to scrambled siRNA alone (n = 3).

To determine the mechanism by which GSCs transition from a quiescent to an activated state, we performed expression profiling of these two populations *in vitro*. Among the genes that showed a change in expression in the BMP- and TGF-β-treated groups was p21. We were particularly interested in this transcript because of its role in mediating activation states in NSCs. In adult mice, p21 has been shown to regulate NSC self-renewal and proliferation, and selected knockout of p21 results in NSC exhaustion^49,50^. Forced expression of p21 results in NSC quiescence, and protects NSCs from senescence^51^. First, using Western blot analysis, we found only minimal expression of p21 in U251 glioblastoma cells in resting-state culture. p21 expression begins to increase within 4 hours of treatment with BMP4, and peaks by 24 hours of BMP exposure (Fig. 7A). The effect of BMP4 on p21 appears to be through induction of transcription at the CDKN1A gene, as demonstrated by increased luciferase activity following U251 transfection with a full-length p21 promoter reporter and treatment with BMP4 (Fig. 7B; n = 3).

We then wished to determine if p21 expression is sufficient and necessary for the anti-proliferative effect of BMP in glioblastoma. To do so, we first examined the effect of p21 inhibition on U251 cell proliferation, using siRNA-mediate knockdown of p21. Knockdown of p21 in U251 cells using siRNA resulted in a negligible increase in U251 proliferation, compared to control, but had a significant effect in mitigating the negative effects of treatment with BMP4 on proliferation (Fig. 7C; n = 3). We then studied the effect of p21 expression on U251 cell proliferation following transfection with a wild-type p21 overexpression vector. Exogenous p21 expression in U251 cells resulted in decreased proliferation, an effect that was not significantly changed by subsequent treatment with BMP4 or TGF-β1 (Fig. 7D; n = 3).

We then sought to elucidate the mechanisms by which p21 inhibits proliferation in glioblastoma. p21 has been shown to bind to and inhibit the oncogenic transcription factor, Stat3, a known target of TGF-β in glioblastoma and driver of GSC self-renewal and proliferation^52,53^. As predicted, coimmunoprecipitation assays demonstrated evidence of p21-Stat3 complex formation in U251 cells treated with BMP4, but not in U251 cells in resting-state culture, which was inhibited by treatment with the chemical p21 inhibitor, sterigmatocystin (Fig. 7E). Our findings demonstrate that p21 binds to Stat3 in U251 cells following activation of the BMP signaling pathway.

Finally, we wished to determine if p21 expression is necessary for BMP to protect glioma cells from temozolomide cytotoxicity. To do so, we generated IC50 curves for temozolomide in U251 cells treated with BMP and scrambled siRNA or BMP and p21 siRNA, compared to scrambled siRNA alone (Fig. 7F; n = 3, p < 0.05). Treatment of U251 cells with BMP4 and scrambled siRNA resulted in a rightward shift of the temozolomide IC50 curve, consistent with a protective effect of BMP4 against temozolomide cytotoxicity in glioma cells. This protective effect was negated by concomitant treatment of U251 cells with BMP4 and siRNA against p21. Our findings support the conclusion that p21 mediates the effect of BMP4 as an inhibitor of cell proliferation and a mediator of temozolomide resistance in glioblastoma.

## Discussion

Taken together, our results demonstrate a subpopulation of quiescent GSCs in glioblastoma, and show that GSC quiescence and activation are mediated by BMP and TGF-β signaling, through the downstream targets, ID1 and p21. These findings speak to the functional heterogeneity of the cancer stem cell identity, and suggest that treatments designed to target CSCs must negotiate differences between these two subpopulations. As in the adult NSC niche, BMP signaling in the glioblastoma microenvironment directs GSCs toward a quiescent state, rather than toward a differentiated, astroglial cell fate, in a process reproducible through expression of p21.

Current studies suggest that cell quiescence is not a passive state, but rather a reversible G_0_ phase functional phenotype that requires active maintenance and regulation, and from which cells may be activated to re-enter the cell cycle^54^. A study of the mouse adult NSC niche of the subgranular zone (SGZ), for example, revealed two groups of adult NSCs: active NSCs that express proliferating cell nuclear antigen (PCNA) and can be identified by a 1-day BrdU pulse, and BrdU-retaining quiescent adult NSCs^55^. The presence of two NSC populations within the NSC niche may speak to the physiologic need to maintain a viable NSC pool: in another study finding that NSC quiescence in the SGZ was maintained by the BMP receptor, BMPR1a, inhibition or ablation of BMP signaling resulted in a temporal increase in progenitor proliferation and subsequent decrease of neurogenesis due to exhaustion of the stem cell pool^56^.

Whether there is a similar need for a quiescent stem-like cell pool to maintain the non-physiologic hierarchy of cancer systems is unclear. Quiescent CSCs have been identified in multiple tumors, including pancreatic adenocarcinoma^57^, breast cancer^58^, liver cancer^59^, and melanoma^60^. Further, studies from multiple cancer systems give evidence to a role for quiescent cells in treatment resistance. Current studies suggest that cell quiescence is not a passive state, but rather a reversible G_0_ phase functional phenotype that requires active maintenance and regulation, and from which cells may be activated to re-enter the cell cycle^54^.

For example, targeting of S phase cells in an *in vivo* liver tumor model using concomitant treatment with a CD13 inhibitor and 5 fluorouracil (5-FU) resulted in a more significant decrease in tumor volume than was seen with treatment with either drug alone^59^. Similarly, JARID1B knockdown to target slow cycling cells in a melanoma animal model results in significant inhibition of tumor growth and progression of tumor metastases^60^. Finally, in an animal model of glioblastoma, treatment with the chemotherapeutic agent, temozolomide, resulted in an enrichment of relatively quiescent stem-like cells, which were able to drive tumor recurrence^2^.

In our study, treatment of GSCs *in vitro* with BMP4 or TGF-β1 directed GSCs toward a quiescent or activated phenotype, respectively. Our *in vitro* studies show that the anti-proliferative effect of BMP4 on glioma cells in mediated by its downstream targets, ID1 and p21. *In vivo*, staining for ID1 identifies a label-retaining cell population in a glioblastoma patient-derived xenograft tumor, supporting the conclusion based on pSmad1 and PCNA co-staining studies in human glioblastoma surgical specimens, that the BMP signaling pathway maintains a quiescent cell population in glioblastoma. Further, BMP signaling exerts a protective effect on glioma cells from temozolomide chemotherapy and radiation, while inhibition of BMP signaling enhances the cytotoxic effects of temozolomide and radiation. Whether glioblastoma cells *in vivo* exhibit functional plasticity in response to cell extrinsic signals as they do *in vitro*, and whether quiescent cell populations contain critical pools of treatment-resistant cells, remains to be elucidated.

### Ethics approval and consent to participate

All studies involving human subjects or tissue were approved by the Research Ethics Board at St. Michael’s Hospital, the Hospital for Sick Kids, and the University of Toronto, or the Institutional Review Board of Northwestern University. All animal studies were performed within the guidelines of an approved Animal Use Protocol at The Centre for Phenogenomics, University of Toronto or Northwestern University.

### Consent for publication

Deidentified patient imaging data have been included in study under the auspices of the Research Ethics Board at St. Michael’s Hospital.

## Methods

### Human samples and study approval

Human glioblastoma specimens and clinical data (including radiographic imaging) were obtained from St. Michael’s Hospital, the St. Michael’s Hospital Brain Tumour Biobank and Clinical Database, and Northwestern Memorial Hospital, following approval by the Institutional Research Ethics Boards of both institutions. Informed consent was obtained from all participants prior to the use of tissue samples or clinical data. All samples were de-identified before analysis. All methods were performed in accordance with the guidelines and regulations of the Research Ethics Boards of St. Michael’s Hospital and Northwestern Memorial Hospital.

### TCGA Analysis

Normalized RNA-expression data on the Affymetrix U133A platform was downloaded from the TCGA data portal. Differentially expressed genes related to TGF and BMP signalling were compared to each subclass of GBM using ANOVA followed by a Post-Tukey analysis. Significance was established at p < 0.05.

### Glioma stem cells and patient-derived xenografts

The GSC lines BT818, BT062508, BT051010, and BT063008 were cultured as neurospheres in Neurocult NS-A Basal Medium (Human Stem Cell Technologies), supplemented with 2mM L-glutamine (Invitrogen), 1X antibiotic/antimycotic (Invitrogen), 1% N2 supplement (Gibco), 2% B27 supplement (Gibco), 75 ng/ml bovine serum albumin (BSA, Sigma), 20 ng/ml human epidermal growth factor (hEGF, Sigma) and 20 ng/ml human basic fibroblast growth factor (hFGF, Sigma).

The GSC lines BT2012035, BT2012087, 101007, 041507, G144, G179, and GliNS1 were cultured and maintained in stem-like media as an adherent monolayer as previously described (Pollard *et al*., Cell Stem Cell 2009). Briefly, these cell lines were cultured in Neurocult NS-A Basal Medium (Human Stem Cell Technologies), supplemented with 2mM L-glutamine (Invitrogen), 1X antibiotic/antimycotic (Invitrogen), 1% N2 supplement (Gibco), 2% B27 supplement (Gibco), 75 ng/ml bovine serum albumin (BSA, Sigma), 20 ng/ml human epidermal growth factor (hEGF, Sigma) and 20 ng/ml human basic fibroblast growth factor (hFGF, Sigma).

NOD *scid* gamma (NSG) mice were purchased from the Jackson Laboratory and maintained in accordance with Toronto Centre for Phenogenomics (TCP) institutional animal protocol. All experimental protocols were approved by the animal utilization committee at TCP. NSG mice underwent stereotactic intracranial injections of 1.5 × 10^5^ BT818 cells into the right corpus striatum, at coordinates 1 mm lateral and 1 mm anterior of the bregma suture, at a depth of 2.5 mm. Following confirmation of tumor formation, mice were treated with EdU and sacrificed at successive time points thereafter.

### Single-cell RNA sequencing analyses

Analysis of scRNA-seq datasets was performed using the R-package Seurat [PMID: 29608179]. The two single-cell datasets were separately analyzed. First, log-normalized single-cell gene expression data was downloaded from 5 patients with primary glioblastoma (*n* = 430 single cells) [PMID:24925914]. The 5,948 genes with highest expression were available for analysis. The most variably expressed genes were determined accounting for the average expression and dispersion of each gene (*n* = 585 genes). Principal component analysis was performed using the most variable genes followed by t-distributed stochastic neighbor embedding (t-SNE) for dimensionality reduction and cluster identification. Assignment of cell type identity to clusters was determined using the genes defined in the single-cell qPCR analyses (*OLIG1*, *BMI1*, *EGFR*), cell cycle marker genes (*TOP2A*, *PCNA*), or pathway-related genes when expression for marker genes were unavailable *(ID3*, *ID2*, *BMP5*, *NES*). Enrichment analysis for gene ontology was performed using the Bioconductor package “topGO”.

A second dataset included scRNA-seq from the same patient at two separate timepoints, the primary tumor (T01, *n* = 27) and recurrent (T01.R, *n* = 16) [PMID: 28263318]. Data were processed as described above. Wilcoxon rank-sum tests were used to assess differential expression between the primary and recurrent tumor.

### Tissue culture

Cells were treated with 20 ng/mL BMP4 (R&D System), 250 ng/mL noggin (R&D System), 20 ng/mL TGF-β1 (R&D System), 3 μM LY364947 (Sigma), and 10 μM BrdU labeling solution (Roche), as described.

### Neurosphere formation efficiency assay

GSCs were plated at various densities (100–2000 cells/mL) in serum-free media and cultured for 7 days. Spheres were defined being of diameter greater than 100 µm. All experiments were performed in triplicate.

### Cell proliferation assay

Cell growth was assayed by plating 5000 cells per well in a TC-treated 96-well clear bottom plate (Corning). Plates were collected at the reported time points and assayed using Alamar Blue as per manufacturer’s protocol. Cell growth was assayed by plating 1 × 10^4^ cells per well in a TC-treated 6-well plate (Corning). Plates were collected at the reported time points and cells were counted using ViCell cell counter.

### Immunohistochemistry

Human glioblastoma specimens and clinical data were obtained from the St. Michael Hospital Brain Tumour Biobank following approval by the Institutional Research Ethics Boards. All samples were de-identified before analysis. Tissue samples were preserved in 10% formalin, dehydrated and embedded in paraffin. Five μm sections were immunostained for pSmad1/5 (Abcam; 1:100), pSmad2 (Cell Signaling; 1:100), or PCNA (Abcam; 1:1000) with antigen retrieval using pressure cooking in 10uM citrate buffer at pH6.0, secondary HRP anti-Rabbit IgG (Vector Laboratories; 1:250), and detection using DAB Substrate kit (Vector lab). Primary antibodies Nuclei were counterstained with DAPI (Sigma; 0.002 mg/ml) for 10 minutes, washed and mounted with Vectashield mounting medium (Vector Laboratories). Images were acquired with a Quorum Spinning Disk Confocal Microscope (Olympus) running Volocity software (Perkin Elmer).

### Western blotting

Cells were washed with cold PBS and lysed. Cell lysates were shaken at 4 °C for 10 min and centrifuged at 14,000 × g for 10 min. Protein concentrations in the supernatants were quantified using the BCA protein assay. Fifteen micrograms of protein were separated on 10% acrylamide/bisacrylamide gels and transferred to PVDF membranes. PVDF membranes and blocked with 5% (w/v) skim milk in PBS/0.1% Tween-20 for 1 h at room temperature. Membranes were blotted with Sox2, Bmi1, nestin, GFAP, Oct3/4, Sall2, Olig1, p21, Stat3, or actin, at 4 °C overnight, then washed and incubated with secondary antibodies (Cell Signaling) for 1 hr at room temperature. Bound antibodies were detected with horseradish peroxidase-linked anti-mouse or anti-rabbit IgG (Cell Signaling), followed by ECL (Amersham). Protein quantification was performed by densitometry (Image Studio, LI-COR). Protein levels were normalized for β-actin.

### Quantitative real-time PCR

Total RNA was extracted from cells in culture using an RNAEasy Kit according to the manufacturer’s instructions (Qiagen), and cDNA synthesis was performed using SuperScript II (Invitrogen). Assays were performed using SYBR Green PCR Master Mix (Applied Biosystems) with a StepOne Real Time PCR System. Predesigned primers were purchased from IDT. Gene transcript levels were calculated using the ΔΔCt method.

### Microarray analysis

Gene expression profiles were measured by Affymetrix GeneChip Human Transcriptome Array 2.0, and RMA normalization was applied. The probe set profiles were summarized to gene level expression profiles by selecting a probe set with maximal IQR (interquartile range) when multiple probe sets were mapped to a gene. Discriminative genes for three groups (BMP4, GSC, and TGF-β1) were identified by Mann-Whitney U-test. The genes in the heat map were selected by Mann-Whitney U-test with a criteria of p-value < 0.05. Gene expression profile for each gene was normalized so that minimal value and maximal value could be converted to 0 and 1, respectively.

### Short interfering RNA knockdown

Predesigned short interfering (si)RNAs targeting human p21 and scrambled control siRNAs were purchased from Qiagen. Two separate siRNAs targeting different sequences within p21 were used to transfect U251 cells using Lipofectamine 2000 (Invitrogen) versus scrambled control siRNA. Cells were harvested 48 hours post-transfection for analysis of protein and RNA levels.

### Statistical analysis

GraphPad Prism 7 software was used to analyze results. Significance was determined by a 2-tailed Student’s t-test.

### Statement of translation significance

Glioblastoma is the most prevalent and aggressive malignant brain tumour in adults, with a median survival following multi-modality therapy of 14.6 months. Recent studies have implicated cancer stem cells within glioblastoma (glioma stem-like cells, GSCs) as mediators of tumor growth, therapeutic resistance and tumor progression. In our work, we characterize the role of the BMP and TGF-β signaling pathways as regulators of CSC state in glioblastoma. We find that BMP pathway activation confers relative resistance to radiation and temozolomide chemotherapy, and defines a quiescent cell population in patients that is enriched by temozolomide chemoradiotherapy. Our study identifies a cellular reservoir for tumor recurrence in glioblastoma following cytotoxic therapy and provides a target to prolong treatment response.

## Supplementary information

Supplemental materials

## Data Availability

Sequence data has been deposited at the European Genome-phenome Archive (EGA), which is hosted by the EBI and the CRG.
