# Supplementary material for: BMP signaling mediates glioma stem cell quiescence and confers treatment resistance in glioblastoma
Source: Sci Rep. 2019 Oct 10;9:14569. doi: 10.1038/s41598-019-51270-1 (PMC6787003; doi:10.1038/s41598-019-51270-1)
Supplement: Supplementary file 1 — Supplemental materials [file 41598_2019_51270_MOESM1_ESM.docx]

**BMP signaling mediates glioma stem cell quiescence and confers treatment resistance in glioblastoma.**

Rohit Sachdeva^1^, Megan Wu^1^, Kevin Johnson^2^, Hyunsoo Kim^2^, Angela Celebre^1,3^, Uswa Shahzad^1,4^, Maya Srikanth Graham^5,6^, John A. Kessler^6^, Jeffrey H. Chuang^2^, Jason Karamchandani^7^, Markus Bredel^8^, Roel Verhaak^2^, and Sunit Das^1,3,4,9^.

1. Arthur and Sonia Labatt Brain Tumour Research Centre, Hospital for SickKids, University of Toronto.

2. The Jackson Laboratory for Genomic Medicine, Farmington, CT.

3. Department of Laboratory Medicine and Pathobiology, University of Toronto.

4. Institute of Medical Sciences, University of Toronto.

5. Department of Neurology, Memorial Sloan Kettering.

6. Department of Neurology and Institute for Stem Cell Medicine, Northwestern University.

7. Department of Laboratory Medicine, Montreal Neurological Institute, McGill University.

8. Department of Radiation Oncology, University of Alabama-Birmingham.

9. Division of Neurosurgery, Li Ka Shing Knowledge Institute, St. Michael’s Hospital, University of Toronto.

Corresponding author:

Sunit Das, MD, PhD

Email: [sunit.das@utoronto.ca](mailto:sunit.das@utoronto.ca)

Phone: 416-864-5548

Fax: 416-864-5596

**Supplementary Materials**

**Supplementary Figure 1.** A) ICC for pSmad1/5 and IDH1-R132H in a human glioblastoma harboring an IDH1-R132H mutation. B) ICC for pSmad2 and IDH1-R132H in a human glioblastoma harboring an IDH1-R132H mutation.

**Supplementary Figure 2.** Heat map of microarray expression data from GSCs treated with BMP4 or TGFβ-1.

**Supplementary Figure 3.** A) ELISA for TGF-β1 in 063008, 101007, and 041507 GSCs, and the fetal neural stem cell line, NSC-1. B) ELISA for TGF-β2 in 063008, 101007, and 041507 GSCs, and the fetal neural stem cell line, NSC-1. C) ELISA for BMP1 in 063008, 101007, and 041507 GSCs, and the fetal neural stem cell line, NSC-1. D ELISA for BMP2 in 063008, 101007, and 041507 GSCs, and the fetal neural stem cell line, NSC-1. E) ELISA for BMP4 in 063008, 101007, and 041507 GSCs, and the fetal neural stem cell line, NSC-1. F) ELISA for BMP7 in 063008, 101007, and 041507 GSCs, and the fetal neural stem cell line, NSC-1.

**Supplementary Figure 4. A)** Expression profile of 19 tumor-propagating cell (TPC)-specific transcription factors (Bernstein GSC panel) in BMP4-treated, TGF-β1-treated and untreated (NT) GSCs. B) TGF-β− and untreated BMP4-responsive target gene expression profile distinguishes TPCs from differentiated glioma cells (DGCs).

**Supplementary Figure 5.** Gadolinium-enhanced, T1-weighted axial magnetic resonance imaging of three patients (**A,B,C**) at initial diagnosis and at early recurrence.

**Supplementary Figure 6.** Gadolinium-enhanced, T1-weighted axial magnetic resonance imaging at initial diagnosis, following adjuvant radiation and concurrent temozolomide therapy, and at frank recurrence.

**Supplementary Figure 7.** A) Survival curve for U251 and U251-TR cells following 48 hours incubation with temozolomide. B) Colony forming assay for U251 and U251-TR cells following 48 hours incubation with temozolomide. C) Proliferation assay for U251 and U251-TR cells. D, E) Representative H&E image of orthotopic xenograft tumors from NOD/*scid* mice implanted with U251 or U251-TR cells.

**Supplementary Figure 1.**


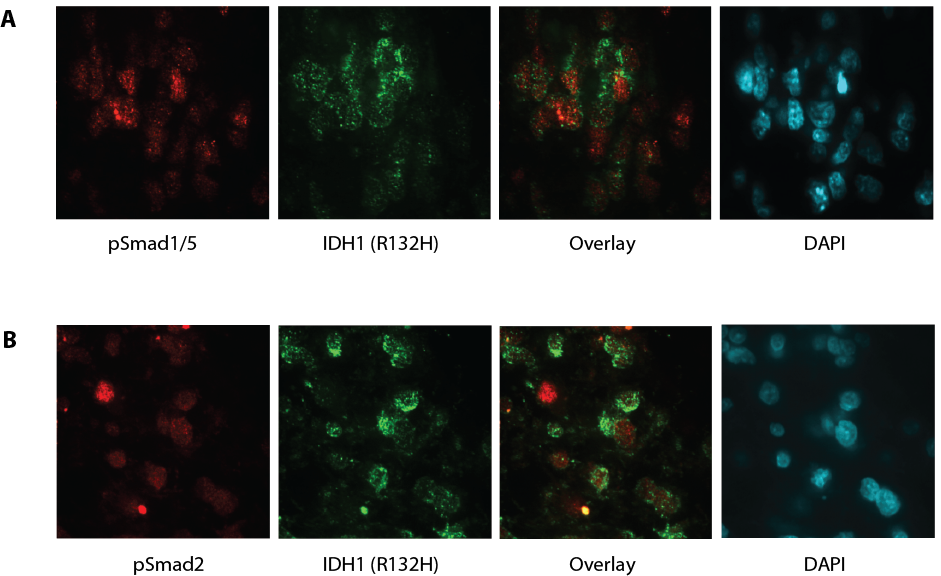


**Supplementary Figure 2.**


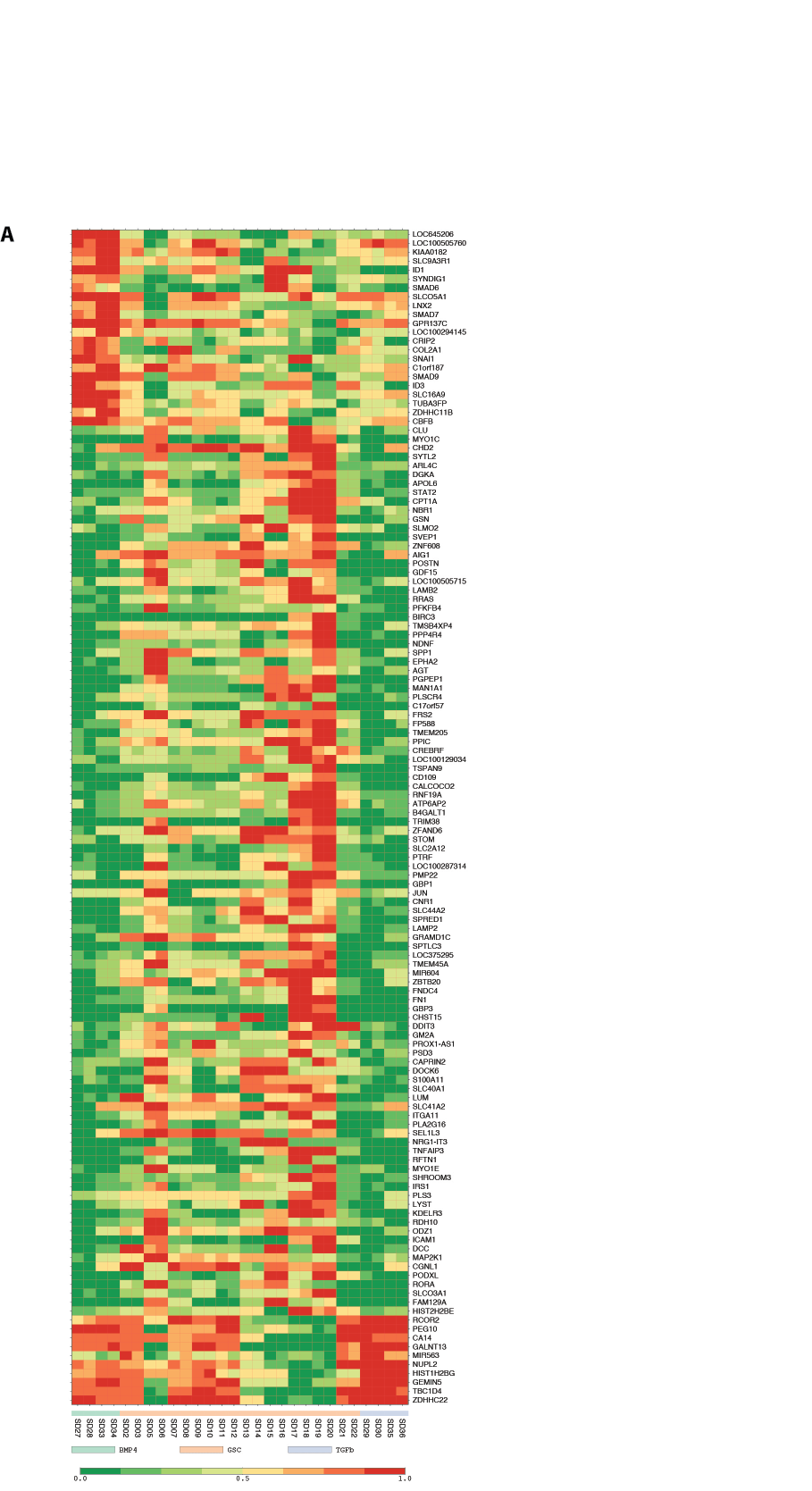


**Supplementary Figure 3.**


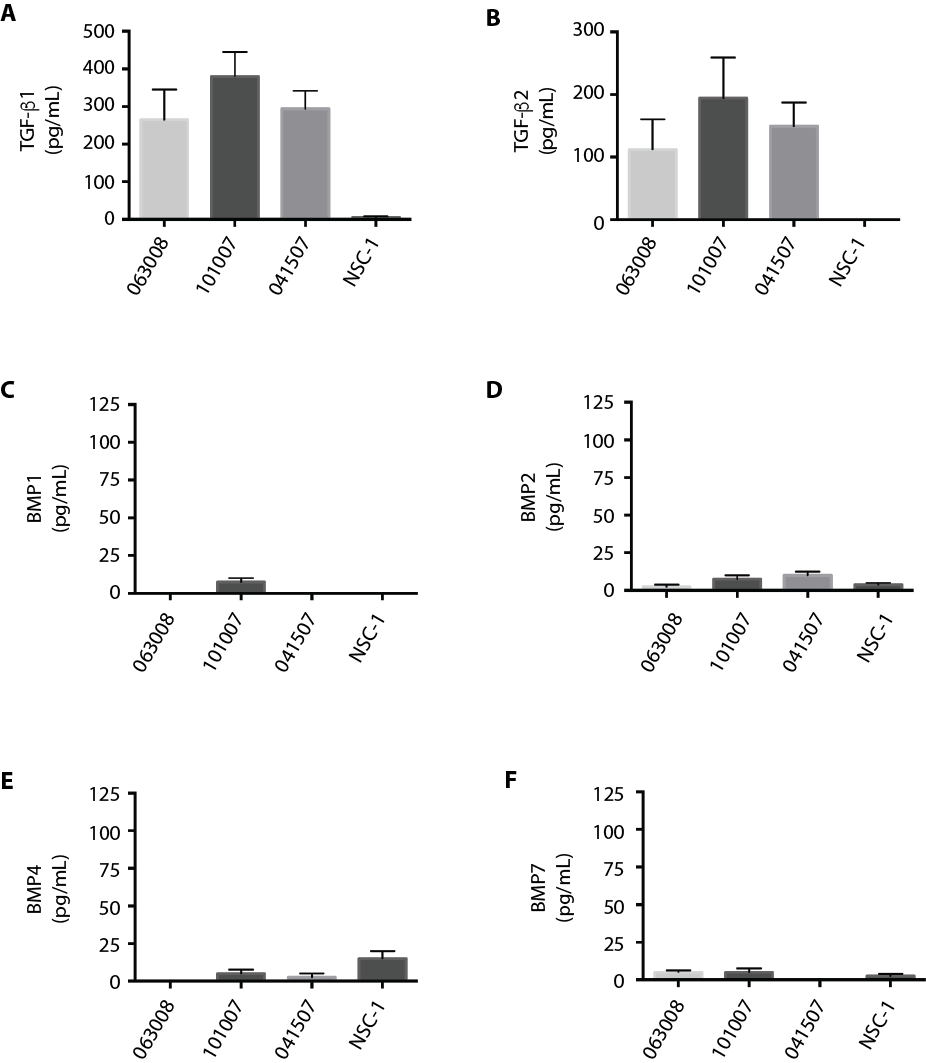


**Supplementary Figure 4.**


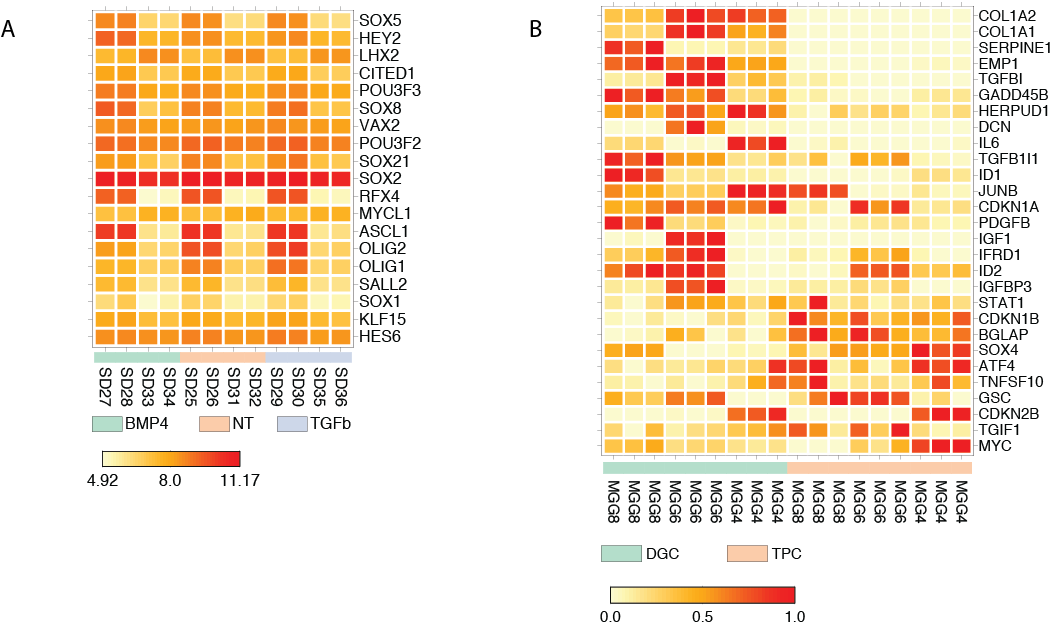


**Supplementary Figure 5.**


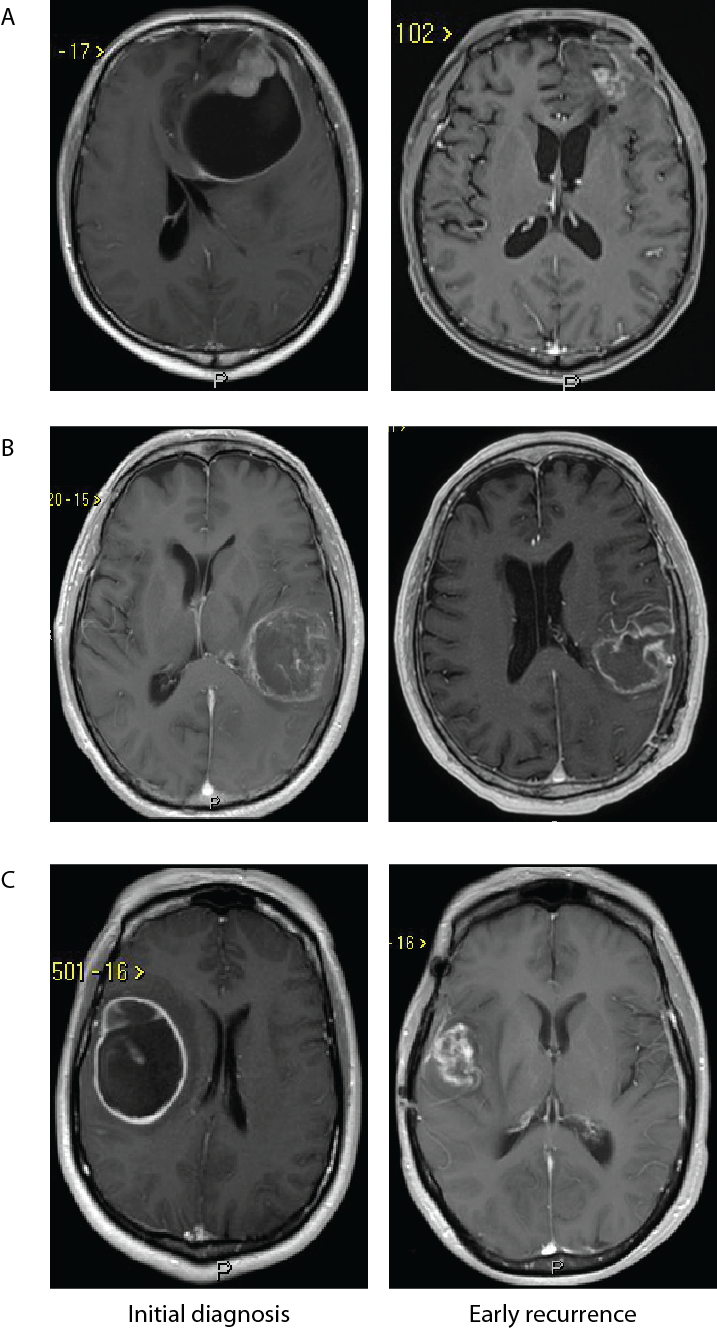


**Supplementary Figure 6.**


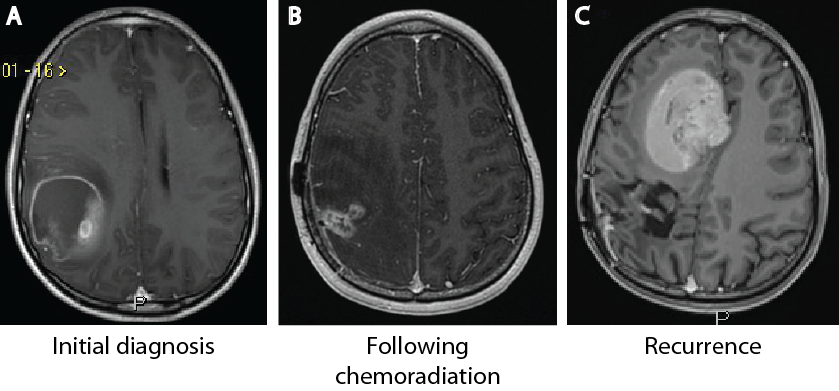


**Supplementary Figure 7.**


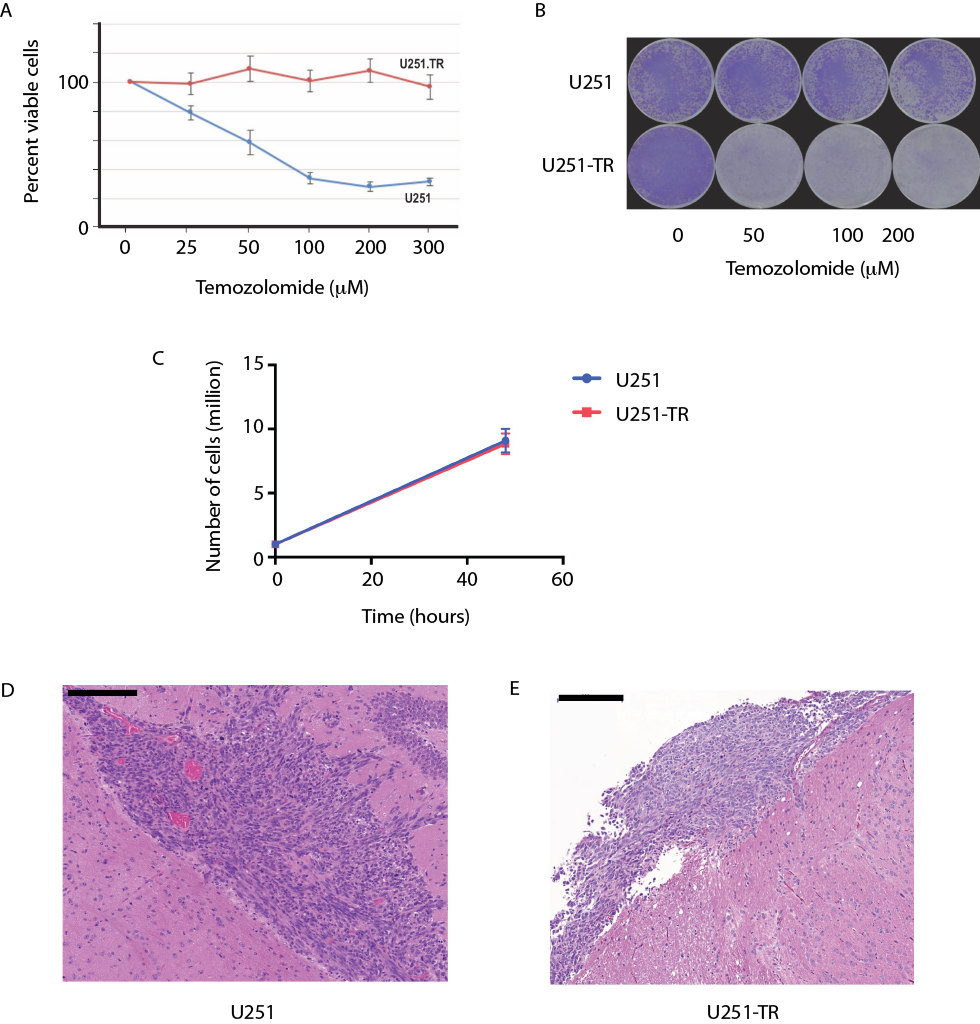


**SUPPLEMENTARY TABLE 1: KEY RESOURCES**

| **REAGENT or RESOURCE** | **SOURCE** | **IDENTIFIER** |
| --- | --- | --- |
| **Antibodies** | | |
| Actin | Cell Signaling | 4967S |
| pSmad1/5 (IHC + ICC) | Cell Signaling | 41D10 |
| pSmad2 (IHC + ICC) | Abcam | ab53100 |
| IDH1 R132H (ICC) | Millipore Sigma | MABC1103 |
| PCNA (IHC) | Cell Signaling | 2586 |
| Sox2 (WB) | Abcam | ab97959 |
| Bmi1 | Abcam | ab38295 |
| GFAP | Abcam | ab7260 |
| Oct3/4 | Santa Cruz | sc-5279 |
| Sall2 | Santa Cruz | sc-135619 |
| Olig1 | Abcam | ab68105 |
| p21 | Cell Signaling | 2947 |
| Stat3 | Abcam | ab5073 |
|  |  |  |
| **Biological Samples** | | |
| Brain blocks (TMA) | St. Michael’s Hospital |  |
|  |  |  |
| **Chemicals, Peptides, and Recombinant Proteins** | | |
| Amaxa electroporation kit | Lonza | VCA-1002 |
| BMP4 | R&D | 314-BP |
| TGF-β1 | R&D | 240-B |
| LY36947 | Selleckchem | S2805 |
| noggin | R&D | 6057-NG |
| Dharmafect | SC Dharmacon | T-2001-02 |
| Matrigel | Corning | 354234, |
| BCA | Pierce Chemical Co. (ThermoFisher) | 23225 |
| PBS | Wisent | 331-010-EL |
| Antibiotic + Antibiotic | Wisent | 450-115-EL |
| N2 Supplement | Gibco | 17502-048 |
| Anti-rabbit HRP | Cell Signaling | 7074S |
| Anti-mouse HRP | Cell Signaling | 7076S |
| DMSO | Sigma | 276855 |
| bFGF | Sigma | F0291 |
| EGF | Sigma | E9644 |
| DMEM | Wisent | 319-005-CL |
| DMEM F12 | Wisent | 319-075-CL |
| B27 | Sigma | 17504044 |
| Penn strep | Wisent | 450-201-EL |
| L-Glut | Wisent | 609-065-EL |
| FBS | Wisent | 920-040 |
| Trypsin/EDTA 0.05% | Wisent | 325-542-EL |
| Accutase solution | Sigma | A6964 |
| Puromycin | Sigma | P8833 |
| Neomycin | Sigma | N6386 |
| Complete mini EDTA free Protease inhibitor cocktail tablets | Roche | 11836170001 |
|  |  |  |
| **Critical Commercial Assays** | | |
| TGF-β1-ELISA | Abcam | ab108912 |
| TGF-β2-ELISA | Abcam | ab100648 |
| BMP1-ELISA | LSBio | LS-F4593 |
| BMP2-ELISA | Abcam | ab119581 |
| BMP4-ELISA | Abcam | ab99982 |
| BMP7-ELISA | Abcam | ab99985 |
| Alarma Blue Cell Viability Reagent | Thermo Fisher Scientific | DAL1025 |
| Edu-FITC | Invitrogen |  |
| sterigmatocystin | Sigma Aldritch | 10048-13-2 |
|  |  |  |
| **Deposited Data** | | |
| Microarray sequencing |  |  |
|  |  |  |
| **Experimental Models: Cell Lines** | | |
| U251 MG | Sigma | 09063001 |
| G144 | Dr. Peter Dirks, Sick Kids |  |
| G179 | Dr. Peter Dirks, Sick Kids |  |
| GliNS1 | Dr. Peter Dirks, Sick Kids |  |
| 818 | Dr. Frederick Lang (MD Anderson Cancer Center) |  |
| BT2012087 | Dr. Sunit Das (UT) |  |
| BT2012035 | Dr. Sunit Das (UT) |  |
| BT062508 | Dr. Sunit Das (Northwestern) |  |
| BT051010 | Dr. Sunit Das (Northwestern) |  |
| BT030909 | Dr. Sunit Das (Northwestern) |  |
| BT063008 | Dr. Sunit Das (Northwestern) |  |
| BT101007 | Dr. Sunit Das (Northwestern) |  |
| BT041507 | Dr. Sunit Das (Northwestern) |  |
|  |  |  |
| **Experimental Models: Organisms/Strains** | | |
| NOD *scid* gamma mice | The Jackson Laboratory | 005557 |
|  |  |  |
| Oligonucleotides | | |
| siRNA p21 | Santa Cruz | sc-29247 |
| siRNA non-target | Dharmacon | D-001210 |
| **Recombinant DNA** | | |
| pGL2-p21 promoter-Luc | Dr. Martin Walsh, Mt. Sinai |  |
|  | | |
| **Software and Algorithms** | | |
| PRISM | Graphpad |  |
| OpenCFU | http://opencfu.sourceforge.net/ |  |
